# Utilization of optical tracking to assess efficacy of intracranial immobilization techniques in proton therapy

**DOI:** 10.1120/jacmp.v16i5.5405

**Published:** 2015-09-08

**Authors:** Wen C. His, Andries N. Schreuder, Omar Zeidan

**Affiliations:** ^1^ Shanghai Proton and Heavy Ion Center Shanghai China; ^2^ Provision Center for Proton Therapy Knoxville TN USA; ^3^ Department of Radiation Physics UF Health Cancer Center‐Orlando Health Orlando FL USA

**Keywords:** optical tracking, interfractional head movement, intracranial immobilization, robotic couch, proton therapy

## Abstract

We present a quantitative methodology to measure head interfraction movements within intracranial masks of commercial immobilization devices used for proton radiotherapy. A three‐points tracking (3PtTrack) method was developed to measure the mask location for each treatment field over an average of 10 fractions for seven patients. Five patients were treated in supine with the Qfix Base‐of‐Skull (BoS) headframe, and two patients were treated in prone with the CIVCO Uni‐frame baseplate. Patients were first localized by an in‐room, image‐guidance (IG) system, and then the mask location was measured using the 3PtTrack method. Measured mask displacements from initial location at the first fraction are considered equivalent to the head interfraction movement within the mask. The trends of head movements and couch displacements and rotation were analyzed in three major directions. The accuracy of 3PtTrack method was shown to be within 1.0 mm based on daily measurements of a QA device after localization by the IG system for a period of three months. For seven patients, mean values of standard deviation (SD) in anterior–posterior, lateral, and superior–inferior directions were 1.1 mm, 1.4 mm, and 1.6 mm for head movements, and were 1.4 mm, 1.8 mm, and 3.4 mm for couch displacements. The mean SD values of couch rotations were 1.1°, 0.9°, and 1.1° for yaw, pitch, and roll, respectively. The overall patterns of head movements and couch displacements were similar for patients treated in either supine or prone, with larger deviations in the superior–inferior (SI) direction. A suboptimal mask fixation to the frame of the mask to the H&N frame is likely the cause for the observed larger head movements and couch displacements in the SI direction compared to other directions. The optical‐tracking methodology provided a quantitative assessment of the magnitude of head motion.

PACS number: 87.55.km

## I. INTRODUCTION

The basic requirement of robust intracranial immobilization techniques^(1)^ is to maintain a fixed spatial correlation between the treated target and the immobilization hardware throughout the course of radiation treatment. Based on clinical evidence and currently available immobilization techniques for brain treatments, a lateral 3 mm margin is typically applied during the treatment planning to account for the setup uncertainty to assure at least 95% of treated patients will fall within this setup uncertainty.^2^, ^3^ To ensure targets are accurately placed at isocenter, patients are positioned using an X‐ray‐based in‐room, image‐guidance (IG) system.^4^, ^5^, ^6^ However, due to geometrical and physical design limitations, X‐ray imaging may not be possible for treatment fields for noncardinal couch angles. For example, a couch treatment position of 45° with respect to beam incidence direction would prohibit acquiring a lateral X‐ray image in the ProCure fixed‐beam line room. In those scenarios, one must trust that the patient anatomy is well immobilized when isocentric couch movements are performed from the setup position to treatment position. For immobilization systems used in proton radiotherapy, the amount of immobilization materials in the proton beam is designed to be minimal to ensure robust beam dosimetry.^(7)^ It leads to overall less rigid^(8)^ immobilization compared to X‐ray therapy immobilization techniques, and subsequently may result in large interfractional target movements within the immobilization device.

The accuracy^9^, ^10^ of robotic couch isocentric movements were found to be within 0.5 mm in radius in our early study^(9)^ with an optical tracking and positioning system (OTPS). If good intracranial immobilization techniques^11^, ^12^ were used with reproducible couch position, the variation of the robotic couch position between treatment fractions should be about 1.0 millimeter after positioning treatment target to isocenter. Using a robotic couch with an interactive MOSAIQ record and verification system (Elekta Inc, Atlanta, GA), the position of the robotic couch for each treatment field at each fraction was recorded after the patient was positioned by IG before and during the proton beam delivery. However, occasional large couch displacements (up to 10 mm in one direction) from reference treatment positions were observed at our institution for some intracranial patients, raising concerns about the quality of fixation of the brain mask to the head frame and the amount of head movements within the mask. To quantify the magnitude of head movements within this proton‐specific intracranial immobilization system, we developed a quantitative methodology using a high‐precision optical tracking system^(9)^ to measure mask locations with respect to the treatment isocenter for each beam position.

## II. MATERIALS AND METHODS

Couch interfraction displacements and rotation from reference (Day 1) position based on daily IG for each treatment field for the three major axes are summarized in Table 1. Five were treated in supine and two were treated in prone. The two commercially available immobilization devices used for the two patient groups are described in the Materials & Methods section A following. To independently investigate the trend of head interfraction movement within the brain mask, a three‐points tracking method, referred to as 3PtTrack, was developed to utilize an optical tracking and positioning system.^13^, ^14^, ^15^ Summary of measured interfraction head motions for each field is shown in Table 1. Details of the OTPS and characterization of robotic couch accuracy can be found in our earlier study.^(9)^ Details of the 3PtTrack method with measurement procedure are described in section B below. Statistical data analysis on studying the trends of couch displacements and head movements within the mask in three major axes are described in section C.

**Table 1:** Summary of measurements performed for each field of the seven patients in this study. Couch displacements and head movements were extracted from MOSAIQ and the 3PtTrack method, respectively. Scored SDs of the couch displacement and head movement for each field are listed in the directions of AP, LAT, and SI. Mean values of SD over all seven patients are listed at the bottom for AP, LAT, and SI directions

*Pt. ID: Setup*	*Fields*	*Fractions*	*# Meas*.	*Couch Disp. (mm)*			*Movement in Mask (mm)*		
				*SD: Z ‐ AP*	*SD: X ‐ LR*	*SD: Y ‐ SI*	*SD: Z ‐ AP*	*SD: X ‐ LR*	*SD: Y ‐ SI*
A1 : supine	Rt Lat	25	10	1.0	0.9	2.0	0.7	1.6	1.6
	Lt Lat	25	10	0.8	1.1	2.3	0.8	1.1	1.2
A2 : supine	Rt Lat	30	12	1.5	2.3	4.3	1.1	1.1	1.7
	Vertex	30	12	1.3	2.2	4.4	1.6	0.9	1.0
A3 : supine	Lt Lat	15	9	0.7	2.4	4.1	1.1	1.7	1.1
	Vertex	15	11	0.7	1.9	4.0	1.3	1.9	1.3
	LSO	15	7	1.0	2.2	2.1	0.8	1.3	1.0
	RSO	15	7	1.8	1.3	2.1	1.1	2.0	2.3
A4 : supine	Vertex	28	15	1.2	1.8	2.9	1.2	1.9	1.3
	RSO	14	8	2.0	1.8	2.9	1.3	0.6	2.3
	LSO	14	7	1.2	1.6	2.8	1.2	0.7	1.9
A5 : supine	RSO	30	13	1.5	1.2	3.5	0.7	1.8	1.5
	RtLat‐Vertex	30	11	0.9	1.6	4.2	1.1	1.2	1.1
B1 : prone	Rt Lat	28	8	1.8	2.3	3.2	0.9	0.8	1.5
	RSO	28	7	1.8	2.3	3.4	1.1	1.4	2.5
	Vertex	28	7	2.0	2.4	2.9	0.8	1.2	0.9
B2 : prone	RPO ‐BST	17	8	1.5	2.1	4.5	1.6	1.3	2.5
	PSO & BST	17	8	1.4	1.7	4.6	1.3	3.6	2.2
	Rt Lat	11	8	1.2	1.6	3.6	1.7	0.9	2.1
	LPO & BST	17	8	2.0	2.4	4.1	1.6	1.2	0.9
			mean of SD	1.3	1.8	3.4	1.2	1.4	1.6

### A. Description of intracranial immobilization techniques

When a patient is immobilized by the mask and the mask/frame is rigidly fixed to the robotic couch, the displacement of couch positions ideally should be within 2.0 mm due to the ±1.5 mm uncertainty of the in‐room IG system^(16)^ and the ±0.5 mm reproducibility of our robotic patient positioning system.^(9)^ The KUKA industrial robot with five rotating joints and three arms was designed by Forte (Forte Automation Systems, Inc., Machesney Park, IL)^(17)^ for patient positioning, and is currently used in all four ProCure Proton Therapy Centers and several other proton centers in the US. In each ProCure center, the Ion Beam Application (IBA)^(18)^ proton beam delivery system was installed with one horizontal fixed beamline, two incline fixed beamlines (300 to vertical axis), and one rotating gantry.^19^, ^20^ The five patients treated in supine are labeled as Group A, and the two patients treated in prone are labeled as Group B. The list of treatment fields for each patient with the number of fraction of each field can be found in Table 1. Couch and mask positions of seven patients were recorded for a total of 21 treatment fields. A total of 186 head movement measurements using the 3PtTrack method were performed on nearly 43% of all treatment fields (n=432), as listed in Table 1

**Figure 1:**
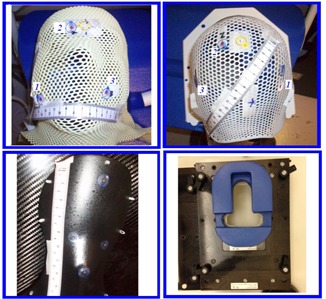
(left) The immobilization systems provided by Qfix for patients treated in supine position. An aqua‐plastic mask (top left panel) was attached by white small pins at the back of the Qfix Base‐of‐Skull (BoS) headframe (bottom left panel). The extended end of the head frame was fixated to the couch by an indexed bar. A MOLDCARE cushion was used as a head rest between the mask and the head frame. (right) The immobilization devices for patients treated in prone position were provided by CIVCO. A type‐S head‐only thermoplastic mask (top right panel) was attached by four locks with guiding pins to the Uni‐frame baseplate with a headrest (bottom right panel). The baseplate was fixated to the couch by an indexed bar. Points were marked on each mask for the three‐points tracking method.

The immobilization devices for Group A patients were provided by Qfix (Avondale, PA). Aqua‐plastic masks were used, as shown in the top left panel of Fig. 1. They were attached by small thin plastic pins at the back of the Base‐of‐Skull (BoS) headframe (Qfix), as shown in the bottom left panel of Fig. 1. The extended end of the BoS headframe was fixated to the couch by an indexed bar. A MOLDCARE cushion (AliMed, Dedham, MA) was used as a headrest between the mask and the head frame. For Group B patients, the immobilization devices were provided by CIVCO (Coralville, IA). A type‐S, head‐only thermoplastic mask, as shown in the top right panel of Fig. 1, was attached by four locks with guiding pins to the Uni‐frame baseplate with a prone headrest, as shown in the bottom right panel of Fig. 1. The baseplate was fixated to the couch by an indexed bar. For both immobilization techniques, the masks were marked with three points that were tracked manually for each beam position using the 3PtTrack method.

### B. The three‐points tracking method using optical tracking and positioning system

A calibration must be applied to transfer measured data by 3PtTrack method to the fixed‐reference system (FRS) frame that is used for the IG system. The in‐room IG software used in this study is VeriSuite by MEDCOM (MedCom GmbH, Darmstadt, Germany). This software regenerates the digitally reconstructed radiograph (DRR) along and perpendicular to the proton beam path of the treatment field during patient positioning. With this approach, VeriSuite has a better capability to estimate the rotations along three major axes in the FRS frame.

**Figure 2:**
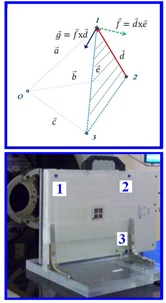
(top) Geometrical principles of the 3PtTrack method. The point “O” is the radiation isocenter of proton beam (i.e., RadIso). (bottom) The daily QA device with three points marked at the flat back plate. Daily displacements of the QA device based on IG localization were measured over a period of three months to evaluate the accuracy of the 3PtTrack method.

During a calibration, two translation movements of couch along two major axes in FRS frame were performed. Based on those two translation movements measured in the camera frame, the rotation matrix and translation vector were obtained to convert measurements in the camera frame to FRS. Details of the calibration procedure and decoding of the rotation matrix can be found in our earlier study.^(9)^ After applying proper calibration, the location of the object was tracked in a FRS frame. In principle, the rotation and displacement of an object can be also directly related to the rotation of a single rigid‐body firmly attached to the object. But, a surgical probe with attached four infrared reflective markers in a unique geometry was utilized to measure the locations of three points marked on the surface of the head mask, as shown in Fig. 1, or on the marked locations on the surface of QA device,^(16)^ as shown in the bottom panel of Fig. 2. The centroid of three points was calculated to determine the location of mask.

The 3PtTrack method utilizes measured three vectors of three points with respect to the treatment isocenter, as shown point “O” in Fig. 2. The treatment isocenter is the same as RadIso (radiation isocenter of proton beam) in our early $\text{publication}.^{\left(9\right)}$ In 3PtTrack method, three vectors $\vec{a},\vec{b}$, and $\vec{c}$ are the locations of three marked points to the RadIso, as shown in Fig. 2. The translation vector of the object is determined by the centroid of the three measured vectors. Two additional vectors are derived as $\vec{d}=\vec{b}-\vec{a}$, and $\vec{e}=\vec{c}-\vec{a}$. A vector perpendicular to the plane of the three points is then derived by $\vec{f}=\vec{d}-\vec{e}$, and a vector perpendicular to $\vec{d}$ in the plane is obtained by $\vec{g}=\vec{f}-\vec{d}$. Three orthogonal unit vectors are obtained as

(1) $$\hat{X}=\frac{\vec{d}}{\left|\vec{d}\right|},\hat{Y}=\frac{\vec{f}}{\left|\vec{f}\right|},\;and\;\hat{Z}=\frac{\vec{g}}{\left|\vec{g}\right|}$$

where *Ẑ* is the cross product of measured *~X* and Ŷ. Finally, the rotation matrix is composed of three unit vectors:

(2) $$R=\left\|\begin{array}{lll} X_{x} & Y_{x} & Z_{x}\\ X_{y} & Y_{y} & Z_{y}\\ X_{z} & Y_{z} & Z_{z} \end{array}\right\|$$

Based on measured initial ($\vec{q};A$) and final ($\vec{r};B$) tracking positions with centroids of three points ($\vec{q};\vec{r}$) and rotating matrixes as shown R in Eq. (2) for (A,B) of a tracked object with the location vector $\vec{L}$ of the RadIso in the camera frame, the displacement $\vec{D}$ between measured initial and final centroids can be calculated by

(3) $$B^{-1}(B(\vec{r}-\vec{L})-A(\vec{q}-\vec{L}))$$

And the rotation C between measured initial and final centroids can be calculated by

(4) $$B^{-1}(\vec{r}-\vec{L})\times A(\vec{q}-\vec{L}).$$

Based on the Euler's rotation theorem, any rotation or sequence of rotations around a fixed point is equivalent to a rotation around a tilted unit vector $\vec{u}$ with its origin at the fixed point. Euler's formula can be expressed by a four‐parameter unit quaternion, where the parameters are a rotation angle and the three rectangular components of unit vector $\vec{u}$. Thus, a six degree‐of‐freedom displacement of three marked points can be presented by a translation of the centroid of three points plus a unit quaternion for the angular rotation of the plane defined by the three points. Therefore, a chain of matrix rotation calculations was converted to a quaternion combination in our optical tracking software. With the fast multiplication of a quaternion combination, the displacement of centroid of three points with the rotation of the plane can be tracked. The tracking described above is still in the camera frame.

The accuracy of 3PtTrack methodology was evaluated by measuring displacements of the QA device over a period of three months. The accuracy of movement for placing QA device by the IG system is ±1.5 mm (1 sigma). Detailed descriptions of the QA device and the daily QA procedure can be found in our publication by Ding et al.^(16)^ Measured displacements by 3PtTrack is compared to the actual distance the device moved from its initial location as determined by the IG system to evaluate the accuracy of the 3PtTrack method.

### C. Analysis of couch interfraction displacement and head interfraction movement

The couch displacements for each treatment field and each fraction over the whole course of treatments were extracted from the MOSAIQ record and verification system. Extracted couch displacements in this study are summarized in Table 1

Although surface‐based OTPSs can be used as an independent and accurate approach to quantify and track any object in treatment room, these systems are rather complex and typically require at least two sets of ceiling‐mounted cameras to generate 3D surfaces. For the 3PtTrack method, a single infrared Polaris Spectra (Northern Digital, Inc, Bakersfield, CA) camera is used to track the locations of three points on a solid surface. Note that three marked points were typically spaced out as much as possible on the mask. As a result, not all of three points for each patient could be directly seen by the camera. By using the long nose of probe, the location of the point outside the camera's direct view could be still measured. A software program was developed to interactively record the location of each point with its own identification number by clicking a wireless mouse when the tip of probe was placed at the point. Visual displacement of each measurement was also developed in the user interface that allows seeing the sequence of measurements, and the locations of the three points were recorded in a database with a time stamp for later study.

For each measurement, the displacement of the mask from its reference position, recorded at Day 1 treatment, was captured in the OTPS control software. Mean values and SDs of measured head movements for each treatment field over the treatment course were calculated in anterior–posterior (AP), lateral (LAT), superior–inferior (SI) directions with respect to patient and present in Table 1. A box‐and‐whisker plot for AP, LAT, and SI directions is used to statistically present measured couch displacements of each field. The box boundaries are the first and third quartiles (25% and 75%) with the band inside the box representing the second quartile (50%; the median), and the ends of the whiskers (horizontal dashes) representing the minimum and maximum values for each field over the treatment course.

## III. RESULTS

### A. Couch interfraction displacements and rotational trends

**Figure 3:**
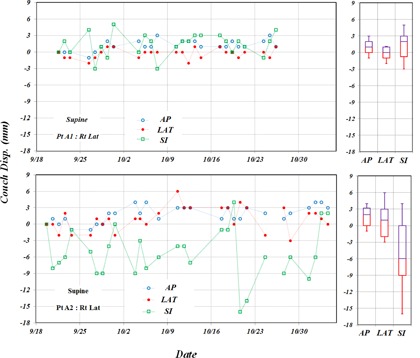
Couch displacements between couch positions recorded at each fraction and the initial setup position are shown for Patient A1 in the top panel and Patient A2 in the bottom panel in three major axes along anterior–posterior (AP), lateral (LAT), and superior–inferior (SI) directions. Both patients were treated in supine position with the Qfix system. Corresponding box‐and‐whisker plots with 1 SD for AP, LAT, and SI are shown in the right panels for each patient.

Couch interfraction displacements for two supine patients are shown in Fig. 3, labeled as A1 (top panel) and A2 (bottom panel). Couch displacements for Patient A1 were in the range of 3 to 6 mm with mean values of 1.2 mm, 0.8 mm, and −0.6 mm, and corresponding SDs of 0.9 mm, 0.9 mm, and 2.0 mm in AP, LAT, and SI directions, respectively. For Patient A2, the mean values of 1.8 mm, 1.0 mm, and −5.2 mm with SDs of 1.5 mm, 2.1 mm, and 4.6 mm in AP, LAT, and SI directions, respectively. A large mean value with large SD in the SI direction indicates that the patient may not have been immobilized well along the SI direction especially for a couple of fractions, where the displacements were more than 10 mm in the SI direction. The distinguished trends of couch displacements between Patients A1 and A2 were seen in terms of mean value and SD. Box‐and‐whisker plots of the three directions for both patient measurements are shown in Fig. 3 for to give an in‐depth statistical representation of the data trends.

**Figure 4:**
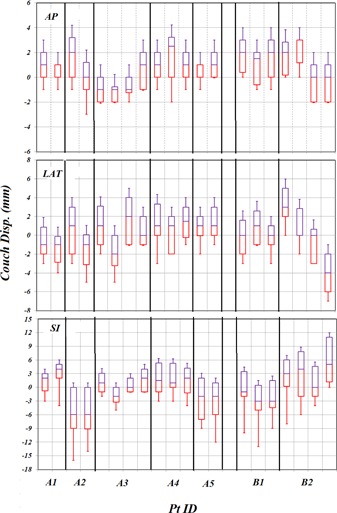
Box‐and‐whisker plots of couch displacements for all patients in each treatment field in AP, LAT, and SI directions from top to bottom panels, respectively.

Figure 4 displays box‐and‐whisker plots of couch displacements for each treatment field over all seven patients. The box‐and‐whisker plots in the AP, LAT, and SI directions are shown in the top, middle, and bottom panels, respectively. Note that the mean value may be biased because it could be largely influenced by the setup at the first fraction as seen in the SI direction of Patient A2 in Fig. 4. Mean values of SDs for the seven patients were 1.3 mm, 1.8 mm, and 3.4 mm for the couch displacement in AP, LAT, and SI directions, respectively. Larger variations of couch displacements in the SI directions are observed as compared to the other two directions; it indicates that couch displacements have directional dependence.

**Figure 5:**
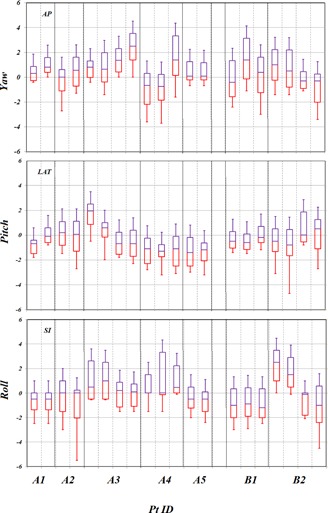
Couch rotations (in degrees) for all patients in each treatment field for yaw about the AP direction, pitch about LAT direction, and roll about SI direction.

To estimate the amount of couch rotational corrections, a summary of recorded couch rotations after patient positioning are shown in a box‐and‐whisker plot in Fig. 5. Measured couch rotations along three axes for each patient were mostly within 3° for the whole treatment with respect to the first fraction. Mean values of SDs of rotation for all seven patients were 1.1°, 0.9°, and 1.1° for yaw about the AP direction, pitch about the LAT direction, and roll about the SI direction, respectively. The mean values of SDs are within 1.0° for measured rotations of QA device. Due to the small corrections in all three rotational degrees of freedom for measured rotations of QA device shown in next section, we consider that quantitative analysis of translational motion only is sufficient to characterize head motion and robustness of the immobilization systems in this study.

### B. The trend of head interfraction movement within mask

**Figure 6:**
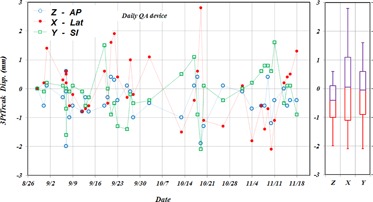
Measured displacements by the 3PtTrack method of the daily QA device in AP, LAT, SI directions with respect to patient with couch position at 270° over a period of three months. The measurements were recorded after the daily QA device was placed in a predefined location by the IG system. SDs are shown in a box‐and‐whisker plot for the three directions.

Measured displacements of the daily QA device by the 3PtTrack method over a period of three months are shown in the top left panel of Fig. 6 in AP, LAT, SI directions with couch position at 270°. All displacements were within 3.0 mm. Mean values of displacements were ‐−0.4 mm, 0.0 mm, and −0.1 mm with SDs of 0.6 mm, 1.1 mm, and 0.8 mm in AP, LAT, and SI directions, respectively. Mean values of measured rotation for QA device were −0.20,−0.10, and −0.20 with SDs of 0.40, 0.20, and 0.20 in AP, LAT, and SI directions, respectively. The mean values and standard deviations of the measurements indicate that the accuracy of the 3PtTrack method is within 1.0 mm in all three directions and can be used to accurately measure the location of brain mask at each fraction.

**Figure 7:**
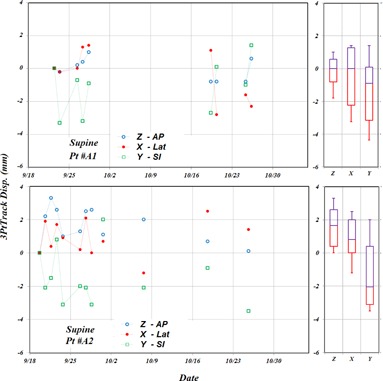
Head interfraction movements within the mask in anterior–posterior (AP), lateral (LAT), and superior–inferior (SI) directions according to measured 3PtTrack displacements for Patients A1 (top panel) and A2 (middle panel). Corresponding mean and SDs for AP, LAT, and SI directions are shown in box‐and‐whisker plots.

Head movement within the mask from its initial location measured by 3PtTrack for Patients A1 and A2 are shown in the top and middle left panels of Fig. 7, respectively. For Patient A1, the head interfraction movements were within 3 mm with mean values of −0.1 mm,−0.3 mm, and −1.3 mm and SDs of 0.6 mm, 1.5 mm, and 1.6 mm in AP, LAT, and SI directions, respectively. The SDs of ∼2.0 mm are close to the accuracy of the IG system for intracranial treatments. For observed large couch displacements of Patient A2, the head interfraction movements were up to 5 mm and resulted mean values of −0.6 mm, 0.9 mm, and −1.5 mm and SDs of 1.0 mm, 1.1 mm, and 1.7 mm in AP, LAT, and SI directions, respectively. A box‐and‐whisker plot is shown in the right panels for each patient for AP, LAT, and SI directions. Results indicate that both patients moved at similar magnitudes within their head masks. Although the mean values were larger for Patient A2, the SDs for both patients were similar, which may indicate the Patient A2 head might not have been positioned correctly inside the brain mask during the initial mask fitting.

**Figure 8:**
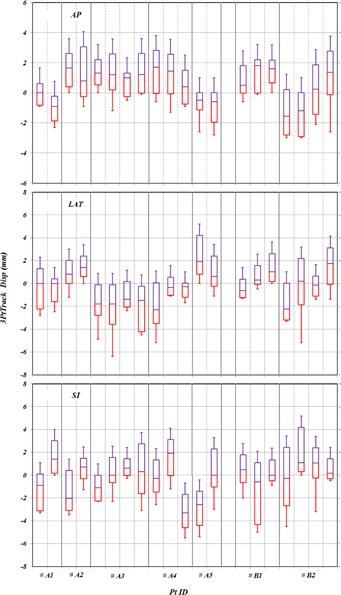
Head movements within the mask, as illustrated by the displacements measured using the 3PtTrack method and shown in box‐and‐whisker plots in each treatment field for all patients.

While the trend of couch movements is significantly different, especially in the SI direction, between A1 and A2, as shown in Fig. 4, the trend of head movements within the brain mask was similar between these two patients. To examine the trend of head movement for all patients, statistical analysis for all treatment fields was performed for all seven patients. The box‐and‐whisker plots in the AP, LAT, and SI directions are shown in the top, middle, and bottom right panels of Fig. 8, respectively. Mean values of SDs for all patients were 1.2 mm, 1.4 mm, and 1.6 mm for couch displacements in AP, LAT, and SI directions, respectively.

## IV. DISCUSSION

The mean value of couch displacements could be biased largely due to setup conditions at the first fraction, as seen in the mean value of couch displacement in the SI direction for Patient A2. Therefore, SD is a better metric for judging trends of couch displacements. While daily variations in mask location are likely caused by head motion, there are other possible causes of mask distortion that can't be easily discerned by our measuring methodology such as hair loss, target shrinkage, tissue swelling, and deformation of the mask itself. When considering all fields for all patients, mean values of SDs in the SI direction were about twice smaller for the head interfraction movement within the mask than for the couch interfraction displacements. The large variations of SDs for couch displacement and head movement were observed in the SI direction with both intracranial immobilization techniques for patients treated in supine and prone. This result indicates that the mask‐to‐head frame fixation was not as rigid as designed for those immobilization systems in the SI direction compared to AP and LAT directions. Large couch displacements with widely varied SDs between patients in the SI direction raised the question on the fixation between the brain mask and headframe and head movement within the mask.

With the accuracy of the 3PtTrack method of 1.0 mm in all three directions, the head interfraction movement within the brain mask could be accurately measured without being influenced by the effects of fixation. The similar trend of measured head movements for two patients with a significantly different trend of couch displacement shows the capability of the 3PtTrack method using the OPTS system to independently quantify head movements within the intracranial mask. Mean values of SDs of head movements within the brain mask for each field were similar for all seven patients, to the order of 1.0 mm in AP and LAT directions, but the mean value was up to 1.6 mm in the SI direction. However, the mean value of 1.6 mm in the SI direction for head movement is still only half of the mean values of couch displacements. Therefore, large couch displacements plus small head movements in the SI direction indicate that the fixation between the brain mask and headframe can be loose in the SI direction.

Although effects of head movement within the mask and deviated fixation of the mask were partially accounted for by daily IG localization procedure, the method developed in this study allows independent evaluation of head movement within the mask. This evaluation provided valuable information for immobilization device vendors to improve individual fixation mechanism to achieve tighter interfraction movement tolerances, and to improve the performance and robustness of immobilization devices.

**Figure 9:**
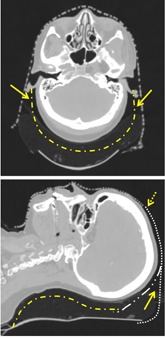
Illustration of the gaps, indicated by arrows, between the MOLDCARE motion and mask in the axial (top) and sagittal (lower) CT views. In the sagittal view at bottom panel, missed mask at superior to the head, as shown by a dot curve, allows the head moving along the SI direction. To improve the conformity on this BoS frame, the dot‐dash curves for a cup shape up to ears in transverse at the axial view and a bended neck section with short length at the sagittal view can reduce the head movements. At the superior end, a cranial flap is necessary to provide better SI support if a shorter length of BoS frame is used as shown dot‐dot‐dash curve at the sagittal view.

Potential causes for head interfraction translational and rotational movements were possibly due to several gaps or voids between the head and the mask, as shown in Fig. 9. Although the mask is effective in restricting head translational movement in the lateral direction, the cup shape of head rest may not be optimal to limit head sliding in the SI direction. In addition, the cup shape of head rest is extended a bit too long toward the superior end of the BoS headframe, as shown in the sagittal view of Fig. 9, which causes lack of conformity between mask and top of the head. Possible solutions to increase contact of mask and superior head is to reduce the length of head rest and to match the curvature of neck by increasing the concave surface on the head rest as shown in the sagittal view of Fig. 9. With the modifications on both LAT and SI directions for the BoS headframe, the head movements within the mask could be reduced. The possibility of reducing head movements of patients treated in prone position needs further investigation.

Improvements on these immobilization techniques may require slight morphing of the posterior, lateral, and superior regions of the overlay shell to make them more pronounced. This will allow for more surface contact of patients outer cranial surface with the mold and overlay shell, and subsequently minimize interfraction cranial motions. Care should be taken to minimize the overlay shell material thickness in the beam path for best dosimetric properties while maintaining its mechanical integrity. Careful choice of the MOLDCARE cushion size for each patient can potentially reduce the size of gaps between mask and cranial surface. In addition, a strict protocol for the preparation of the mask and MOLDCARE should be followed to allow for an overall relatively uniform a reproducible immobilization setup for all patients. Increased confidence in the quality of intracranial immobilization techniques may reduce the need for frequent imaging within each treatment fraction to verify patient positioning for each beam and, hence, decrease overall treatment time and increase patient comfort without sacrificing localization accuracy.

## V. CONCLUSIONS

An optical tracking and position system (OTPS)‐based technique, 3PtTrack method, was developed in‐house to directly measure the position of intracranial masks for each treatment field. The difference in measured couch and mask positions in space allowed for quantification of patient interfraction motion within the mask. By studying the actual causes of head movements within masks, we propose a modification to the immobilization device to reduce head movements within the mask and to address the poor fixation between brain mask and headframe. Confidence in the quality of intracranial immobilization techniques may require less imaging, which could reduce the treatment time for brain cancer. This method can also be utilized to assess the efficacy of any immobilization device for different disease sites.

## ACKNOWLEDGMENTS

The authors would like to thank the radiation therapy technologist team at the ProCure Proton Therapy Center in Oklahoma City for providing assistance with this project.
